# Psychometric Properties and Measurement Invariance of the English Version of the Satisfaction with Life Scale (SWLS) for Non-Native English Speakers

**DOI:** 10.3390/ejihpe14060113

**Published:** 2024-06-12

**Authors:** Giusy Danila Valenti, Palmira Faraci

**Affiliations:** 1 Department of Psychology, Educational Science and Human Movement, University of Palermo, 90128 Palermo, Italy; 2 Department of Human and Social Sciences, University of Enna “Kore”, 94100 Enna, Italy; palmira.faraci@unikore.it

**Keywords:** life satisfaction, Satisfaction with Life Scale, measurement invariance, cross-cultural

## Abstract

This cross-cultural study seeks to (a) investigate the internal structure of the English version of the Satisfaction With Life Scale (SWLS) when administered to non-native English speakers (i.e., Asian individuals and (b) test for measurement invariance with its Italian counterpart). The whole sample comprises 338 participants, including 167 Asian international university students residing in the United States (50.3% females; *M_age_* = 23.82, *SD* = 3.78) and 171 Italian university students living in Italy (69.6% females; *M_age_* = 22.38, *SD* = 4.24). The dimensionality of the scale is assessed through confirmatory factor analysis (CFA), and multi-group confirmatory factor analysis (MG-CFA) is employed to examine measurement invariance. The results confirm the one-dimensionality of the SWLS [χ^2^ = 9.815; *df* = 5; CFI = 0.989; TLI = 0.977; SRMR = 0.027]. Furthermore, achieving full strict invariance indicates that the SWLS items exhibit similar structures across both samples. The scale shows satisfactory internal reliability (α = 0.863, ω = 0.866). Overall, this study supports the cross-validity of the English version of the SWLS and underscores its robustness and suitability for assessing life satisfaction among non-native English speakers.

## 1. Introduction

Life satisfaction, a crucial component of subjective well-being (SWB), pertains to individuals’ conscious evaluation of their own lives in relation to self-imposed standards [1]. When people assess their life events against personal criteria, they tend to report higher levels of life satisfaction. Empirical findings have emphasized the fundamental role played by life satisfaction in various aspects of psychological and physical well-being. For instance, as reported by Pavot and Diener [2], life satisfaction was predictive of greater social skills and stronger social relationships, while reducing suicidal risk. Garrido et al. [3] and Habibov et al. [4] highlighted a bidirectional relationship between life satisfaction and several health-related quality of life outcomes, such as human functioning, vitality, mental health, and social functioning, and stressed that such associations were heterogeneous, based on gender and age. Following this, Milas et al. [5] reported that individuals lower in life satisfaction tend to cope with stressful life events by performing maladaptive behaviors, such as avoidance and withdrawal, as well as using alcohol or drugs. Also, Szcześniak et al. [6] showed that people who perceive their own lives as satisfying are more likely to have increased self-confidence and self-efficacy, providing evidence that life satisfaction has a great impact on individuals’ overall sense of worth and value, thus affecting their way of interacting with themselves and with others. Furthermore, Haraman [7] indicated that students who are satisfied with their own lives are more prone to develop a greater level of achievement motivation, suggesting that life satisfaction may also have positive influences in the academic area. Therefore, due to these implications and their associations with several positive life outcomes, research on life satisfaction has flourished across diverse fields. 

To measure the cognitive aspect of SWB, Diener et al. [1] developed the Satisfaction with Life Scale (SWLS), a concise unidimensional instrument comprising five items that capture overall life satisfaction. Interestingly, Pavot et al. [8] found that SWLS items address two distinct temporal dimensions of life satisfaction: present (Item 1, Item 2, and Item 3) and past (Item 4 and Item 5). This observation suggests that a two-factor structure for the questionnaire might be appropriate. From this perspective, Busseri et al. [9] proposed that individuals’ reported life satisfaction levels are influenced by the temporal context of their evaluation. However, despite some studies’ support for a two-factor solution for the SWLS (albeit they did not find it significantly better than the one-dimensional model), the strong correlation between factors (*r* > 0.80) led researchers to reject it as the optimal representation of the data [10,11]. Consequently, the prevailing consensus in the literature favors the one-dimensionality of the SWLS as its underlying structure. In summary, understanding life satisfaction and its measurement remains a critical area of investigation, with implications for both research and practical applications. 

The SWLS has undergone extensive validation since its inception. Translated into more than 35 languages (see http://labs.psychology.illinois.edu/~ediener/SWLS.html, accessed on 13 January 2024), the SWLS has been widely applied across diverse contexts, including studies involving adolescents, adults, the elderly, and both healthy and clinical individuals. Researchers have meticulously examined the psychometric properties of the scale, encompassing assessments of internal consistency, test–retest reliability, construct validity, and structural validity. However, the study examinations of the SWLS have predominantly been explored among native speakers of the specific SWLS adaptations employed. The current study addresses this gap by examining the psychometric properties of the English version of the SWLS when administered to non-native English speakers. Such an investigation could be useful in providing additional knowledge about the conceptualization and evaluation of life satisfaction across languages and cultures.

### Measurement Invariance of the SWLS across Countries: Main Previous Findings

Research has rigorously examined the equivalence of SWLS items across various sub-groups. These investigations have spanned demographic factors [10,11,12,13,14,15,16,17,18], temporal consideration [12,19], and cross-cultural contexts [13,14,15,20]. A critical consideration in psychometric evaluation is measurement invariance (MI). When comparing mean scores across different groups or repeated measurements, the underlying construct’s equivalence becomes pivotal. Without MI, such comparisons may lack meaningful interpretation or even yield misleading results. In cross-cultural research, investigating MI serves as a fundamental prerequisite before embarking on any study, ensuring more interpretable and robust findings. 

Of particular interest is the cultural sensitivity inherent in evaluating life satisfaction. Given that the SWLS comprises items capturing global cognitive judgments about life, it is reasonable to hypothesize that cultural nuances may influence individuals’ interpretations of these items. Consequently, assessing the invariance of SWLS items across specific cultural groups becomes a necessary and fundamental investigation. The SWLS comprises items that capture global cognitive judgments about life, reflecting culturally specific notions of a “good life”. Therefore, it is theoretically plausible to anticipate cultural variations in the evaluation of quality of life. Prior research has indeed highlighted differences between Western and Eastern countries in the conceptualization of various life outcomes [20,21]. These disparities may stem from divergent perspectives on quality of life, influenced by individualistic versus collectivistic cultural orientations. 

Given this backdrop, it is reasonable to hypothesize that individuals from different cultural backgrounds may perceive SWLS items differently and interpret response scales in distinct ways. As a consequence, measurement non-invariance across countries may emerge, emphasizing the need for a rigorous examination of the SWLS’s cross-cultural applicability. Therefore, understanding the cross-cultural measurement properties of the SWLS is essential for meaningful and valid assessments of life satisfaction across diverse populations. 

Cross-cultural investigations have revealed notable differences in response styles, particularly concerning extreme responses. Individuals from Western countries tend to exhibit a higher propensity for extreme responses when evaluating subjective constructs. Intriguingly, response patterns also appear to be influenced by language. Specifically, respondents using their native language are more likely to provide extreme responses, whereas those completing English-language questionnaires tend to favor middle-of-the-scale responses [22]. 

MI analyses across diverse cultural contexts have yielded mixed findings. Noteworthy examples include the following: (1) Esnaola et al. [13] reported strict MI for the SWLS between Spanish and Mexican populations; (2) Whisman and Judd [20] evidenced partial scalar invariance across individuals from U.S.A., England, and Japan; (3) Jang et al. [23] evaluated MI of the SWLS in a multinational context, 26 countries, revealing that configural and metric MI were upheld, whereas scalar MI was not fully supported (similar results were corroborated in a recent study [14]); (4) Zanon et al. [15] did not find evidence of MI when examining the psychometric properties of the SWLS in Brazilian and U.S.A. university students; (5) Schnettler et al. [24] encountered similar non-invariance findings in their study involving Ecuadorian and Chilean individuals; (6) Jovanović et al. [25] have recently investigated MI of the SWLS across adolescents residing in 24 countries, revealing complexities in achieving full invariance. Hence, understanding the cultural nuances in life satisfaction assessment is essential for valid cross-cultural comparisons. Thus, the findings regarding MI analyses for the SWLS suggest that additional studies on this topic are needed; furthermore, considering multiple potential cultural sources of non-invariance in life satisfaction evaluation is highly desirable. 

The present study has a dual objective. First, we aim to evaluate the psychometric properties of the English version of the SWLS when administered to non-native English speakers, specifically individuals from Asian backgrounds. This assessment encompasses considerations of structural validity and internal consistency. Second, we extend our inquiry to encompass MI, examining the equivalence of the SWLS between the English and Italian versions. Notably, this study introduces a novel approach by simultaneously considering two potential sources of non-invariance in psychological assessment. First, we explore how the country of origin may influence the conceptualization and evaluation of life outcomes. Second, we investigate how respondents’ mother tongue might impact their response style. Ultimately, this cross-cultural investigation contributes to a deeper understanding of the SWLS’s applicability across linguistic and cultural contexts, shedding light on critical aspects of subjective well-being assessment.

## 2. Materials and Methods

### 2.1. Sample and Data Collection Procedure 

MI analyses are sensitive to the sample sizes of compared groups, necessitating almost equivalent group sizes [26]. In this study, we carefully selected approximately equal-size samples from two distinct cultural contexts to explore the psychometric properties of the SWLS. 

Sample 1 was composed of 167 Asian university students (50.3% females; *M_age_* = 23.82, *SD* = 3.78) enrolled in a U.S. university. Recruitment occurred through informal contacts across campus locations (libraries, restaurants, shops). English served as the language of communication. Participants completed the SWLS questionnaire in a paper-and-pencil format. Inclusion criteria included perceived English proficiency, with participants self-assessing their proficiency as at least fair. We asked participants to answer a single-item measure, “How good do you think your overall English language ability is?”, by selecting one of four options: “poor”, “fair”, “good”, or “excellent”. However, no Asian students identified themselves as having poor perceived English proficiency, resulting in the inclusion of all participants in subsequent analyses. Missing data were addressed using mean imputation, a suitable method for cases missing completely at random (<5% of data; [27]). Sample 2 comprised 171 Italian university students (69.6% females; *M_age_* = 22.38, *SD* = 4.24) attending a psychology course. Data collection occurred during class sessions, with students responding to SWLS items online. 

Participants received assurances of voluntary participation (without monetary reward) and confidential data treatment for scientific purposes. An information sheet provided background details and study purpose, allowing participants to seek clarification. The research study posed no legal, social, political, economic, or health risks to the participating individuals. Participants provided informed consent by voluntarily completing the questionnaire (combined verbal and implied consent). Ethical approval for the study was obtained from the University of Enna “Kore” (code: 24918). All procedures adhered to the principles outlined in the Declaration of Helsinki.

### 2.2. Instrument

For Sample 1, we employed the original version of the SWLS [28]. For Sample 2, we utilized the Italian-adapted version of the instrument [29], which confirmed the one-factor structure of the original scale, with good internal reliability and concurrent validity. The SWLS comprises five items, rated on a 7-point Likert-type scale (ranging from 1 = strongly disagree to 7 = strongly agree), designed to assess overall life satisfaction. Higher scores indicate greater life satisfaction.

### 2.3. Data Analyses 

Prior to the main analyses, we examined invariance related to demographics (age/gender). Ensuring sample invariance across groups is crucial, as it provides confidence that observed differences are genuinely reflective of cultural variations rather than demographic dissimilarities. Specifically, gender invariance was tested using χ^2^, whereas age invariance was tested through an independent samples *t*-test. Descriptive statistics (means, standard deviations, skewness, kurtosis, and inter-item correlations) were computed for SWLS items within each group. Univariate normality was evaluated based on skewness and kurtosis (deviations exceeding |1| and |3|, respectively). Multivariate normality assumptions were verified using Mardia’s test, comparing results with the critical chi-squared value associated with *p <* 0.001 [30].

In this study, we conducted independent confirmatory factor analyses (CFA) on the two distinct subsamples to assess the dimensionality of the scale and gather validity evidence for each group. Traditional goodness-of-fit indices were employed to evaluate model fit, including the comparative fit index (CFI), the Tucker–Lewis index (TLI), the standardized root mean square residual (SRMR), and the root mean square error of approximation (RMSEA). According to the established guidelines [31,32,33], CFI and TLI values exceeding 0.95 indicate an adequate fit. Additionally, SRMR and RMSEA values below 0.08 or 0.05 indicate acceptable and excellent model fit, respectively. Drawing from prior research on the structural dimensionality underlying the SWLS [13,14,17,23], we specified a one-dimensional model as the fundamental structure (although a two-factor structure for the SWLS was supported in some studies, we did not test such a model, as the minimum recommended number of observed indicators for each latent variable was not met). Internal consistency was assessed using McDonald’s ω and Cronbach’s α, with values exceeding 0.70 indicating good reliability [34].

The assessment of MI for the SWLS was performed within the framework of multi-group confirmatory factorial analysis (MG-CFA). This approach involves comparing models with progressively increasing levels of restrictions. We tested four types of MI: (a) *configural invariance* (Model 0); (b) *metric invariance* (Model 1); (c) *scalar invariance* (Model 2); and (d) *strict invariance* (Model 3). Specifically, Model 0 is an unconstrained model, in which parameters are allowed to vary freely, and it serves as the baseline model for subsequent comparisons within the invariance hierarchy; in Model 1, factor loadings are constrained to be equal across groups; in Model 2, item intercepts are constrained to be equal across groups; Model 3 assumes that the measurement error in the manifest indicators is the same across groups. Each constrained model was nested within a less restricted one. To evaluate model invariance, we employed two key indicators: a change in comparative fit index (ΔCFI) below −0.010; and a change in root mean square error of approximation (RMSEA) below 0.015. These criteria align with the established guidelines [35,36] and provide for comparisons between subsequent models. Each level of MI requires evidence supporting invariance at the prior level (e.g., strict MI entails equivalence of residual variances, item intercepts, loadings, and factor structure). If Model 2 or Model 3 did not receive support, we iteratively relaxed constraints on factor loadings (for Model 2) or item intercepts (for Model 3) until achieving a partially invariant model.

## 3. Results

### 3.1. Preliminary Data Screening

Examination of demographics distribution revealed non-equivalence between the two subgroups concerning gender [χ^2^_(1, 338)_ = 13.107, *p* < 0.001] and age [*t*_(336)_ = 3.296, *p* < 0.001]; specifically, Italian participants were slightly younger (*M_age_* = 22.38, *SD* = 4.24) compared to the Asians (*M_age_* = 23.82, *SD* = 3.78) and were mostly females (69.6%), whereas the Asian sample was equally balanced across genders (50.3% females). The distinct gender and age compositions across the two subsamples may impact the MI of SWLS items. As depicted in Table 1, both versions of SWLS exhibited univariate and multivariate normal distribution. Skewness and kurtosis values fell within the suggested thresholds. The Mardia’s coefficient was below the critical value (48). Thus, the maximum likelihood (ML) estimation method was adopted. Adequate inter-item correlations (*r* > 0.35 in both samples) indicated that each item significantly contributed to life satisfaction assessment (see Table 1 for an overview and Table A1 in Appendix A for detailed inter-item correlations for both SWLS versions).

### 3.2. Factor Structure and Reliability of the SWLS 

Both SWLS versions adequately supported the traditional one-dimensional structure of the scale. Excellent fit indices were reported. All items significantly contributed to life satisfaction assessment, with factor loadings exceeding 0.543. Coefficients of internal reliability further supported the suitability of the one-dimensionality (see Table 1).

### 3.3. Measurement Invariance (MI) of the SWLS

Following the identification of the baseline model, we systematically examined the equivalence of this model across the two distinct subgroups. This investigation involved progressively imposing more stringent constraints to evaluate the models fit. 

Table 2 presents the goodness-of-fit statistics for tests of MI within the one-dimensional model. Notably, the addition of more restrictive constraints did not result in substantial decrements in model fit. Specifically, the changes in comparative fit index (ΔCFI) and root mean square error of approximation (ΔRMSEA) remained below the proposed cut-off points.

Consequently, the more parsimonious (invariant) model was retained. Based on these findings, we conclude that the set of equivalences holds. Therefore, the two versions of the SWLS may be confidently regarded as equivalent across the studied subgroups. Indeed, the achievement of the highest level of MI hierarchy provides confidence that the group mean differences on the scale scores are driven by real group differences and not by other factors; thus, the SWLS scores between these two cultural groups can be adequately compared to each other.

## 4. Discussion

The present cross-cultural study contributes to the extensive literature on the psychometric properties of the SWLS, a widely used assessment tool for evaluating the cognitive aspect of global SWB. As a novel contribution, this research investigates the psychometric properties of the scale when administered to non-native speakers. Specifically, we examine the structural validity and internal consistency of the English version of the SWLS among Asian university students residing in the USA, while also assessing its equivalence with the Italian version. Previous studies have highlighted potential differences in adaptive outcomes between Eastern and Western cultures [20,21], as well as variations in response styles based on used language (mother tongue or L2) [22]. Our study explores whether these culture-related factors influence the conceptualization and evaluation of life satisfaction. Given our multicultural society, characterized by diminishing boundaries and increasingly diverse populations, robust psychometric measures for non-native speakers are essential for a comprehensive understanding of cultural similarities and differences. 

First, our findings support the well-established one-dimensional model of the SWLS, consistent with prior research [10,12,14,17,29,37]. The one-single-factor solution demonstrates adequacy for both subsamples. Both versions of the SWLS demonstrate an adequate level of internal consistency, as reliability coefficients meet commonly accepted rules of thumb. Our findings reveal strict invariance, indicating that SWLS items maintain consistent scale structures across all participants. This result is noteworthy given potential sources of non-equivalence, including language-related factors and demographic composition (age and gender) within subgroups. 

In an era characterized by globalization and digitalization, where academic and professional mobility from East to West is commonplace, traditional cultural influences may impact individuals’ perceptions, conceptualizations, and evaluations of life outcomes to a lesser extent. The achievement of strict invariance suggests a shared understanding of life satisfaction, transcending cultural backgrounds. Our results contribute to cross-cultural research by affirming the suitability and reliability of the English version of the SWLS for assessing subjective well-being (SWB) among Asians residing in English-speaking countries. This finding holds particular relevance in multicultural societies, such as the U.S.A., where the Asian population continues to grow steadily. 

### Limitations and Suggestions for Future Works

Several limitations warrant consideration in the context of our study. First, the administration of the English version of the SWLS was limited to Asian university students, thereby restricting the generalizability of our findings. To enhance the robustness of the SWLS, future research should involve individuals from diverse cultural backgrounds and linguistic contexts. Moreover, both study groups consisted exclusively of university students, which raises questions about the applicability of our results to the broader population. Also, gender and age non-equivalence between the two groups may represent a further concern for the current study. The relatively small sample sizes within each subsample also constrained our ability to employ further robust analytic techniques, such as item response theory (IRT), which may significantly improve the measurement accuracy of the scale and provide additional details about item parameters (such as item difficulty and item discrimination). As a further concern, our study did not include native English speakers for the English version of the SWLS. This may have been useful for providing a link between the two involved samples (Asians vs. Italians) and for minimizing any potential biases associated with their different and specific characteristics, which might have made the comparison somewhat questionable. 

In light of these limitations, we recommend that future investigations address the following areas: (1) further inspection of the psychometric properties of the English version of the SWLS is essential (this examination should encompass aspects such as reliability, validity, and factor structure); (2) comparative analyses involving SWLS versions beyond the Italian one are warranted (investigating measurement invariance across different language versions will enhance the understanding of its cross-cultural applicability); (3) to broaden the scope of our findings, future studies should include a more diverse sample drawn from the general population. 

Testing the psychometric properties of scales assessing related psychological constructs among non-native speakers will contribute to a more comprehensive understanding of well-being assessment. Addressing these limitations will advance our knowledge of the SWLS and its utility in evaluating life satisfaction across diverse populations.

## 5. Conclusions

The present study contributes to the existing literature by examining the psychometric properties of the Satisfaction with Life Scale (SWLS) within a cross-cultural context. Specifically, we focus on non-native speakers, investigating the structural validity and internal consistency of the English version of the SWLS administered to Asian university students residing in the U.S.A., while also assessing its equivalence with the Italian version. 

Despite the acknowledged limitations, our examination of the SWLS’s psychometric properties provides compelling evidence of its robustness and underscores its suitability for assessing cognitive SWB beyond conventional cultural boundaries. Cross-cultural research necessitates the use of psychometrically sound measures to evaluate various life outcomes. Such measures enable meaningful and interpretable comparisons of mean scores across different versions of the same scales, particularly when administered to individuals from diverse cultural backgrounds. 

In this context, the English version of the SWLS shows worthy qualities, even when applied to non-native English speakers. This finding holds particular significance for contemporary cross-cultural research. The availability of robust psychometric scales suitable for individuals with different mother tongues streamlines assessment procedures, especially when dealing with multicultural samples. 

In conclusion, our study underscores the importance of robust psychometric measures for non-native speakers, contributing to a broader understanding of cultural similarities and differences in the assessment of life satisfaction. As our global society continues to evolve, such measures facilitate meaningful cross-cultural comparisons and enhance our understanding of individual well-being.

## Figures and Tables

**Table 1:** Descriptive statistics and reliability coefficients of the SWLS.

Item	English Version of the SWLS for Non-Native English Speakers (*n* = 167) ^a^					Italian Version of the SWLS (*n* = 171) ^b^				
	λ	*M*	*SD*	S	K	λ	*M*	*SD*	S	K
SWLS_1	0.805	4.70	1.37	−0.428	−0.533	0.846	4.50	1.52	−0.630	−0.659
SWLS_2	0.754	4.92	1.31	−0.494	−0.567	0.746	4.28	1.55	−0.432	−0.791
SWLS_3	0.922	5.02	1.30	−0.571	−0.230	0.915	4.58	1.48	−0.726	−0.237
SWLS_4	0.727	4.84	1.43	−0.583	−0.436	0.669	4.42	1.48	−0.369	−0.618
SWLS_5	0.612	3.94	1.77	−0.105	−1.152	0.543	3.57	1.82	0.378	−0.970
Mardia’s coefficient	34.47					39.79				
Inter-item correlations	0.426 *** < *r* < 0.733 ***					0.324 *** < *r* < 0.725 ***				
ω	0.866					0.857				
α	0.863					0.851				

Note: λ = factor loadings; M = mean; SD = standard deviation; S *=* skewness; K = kurtosis. ^a^ χ^2^_(*df*)_ = 9.815_(5)_; CFI = 0.989; TLI = 0.977; RMSEA = 0.076, 90% CI (0.000–0.146); SRMR = 0.027. ^b^ χ^2^_(*df*)_ = 9.235_(5)_; CFI = 0.990; TLI = 0.979; RMSEA = 0.070, 90% CI (0.000–0.141); SRMR = 0.028. *** *p <* 0.001.

**Table 2:** Measurement invariance of the SWLS.

Model ^a^	χ^2^	*df*	Δχ^2^	Δ*df*	*p*	TLI	CFI	ΔCFI	RMSEA (90% CI)	ΔRMSEA	SRMR
M0	19.050	10	-	-	0.040	0.978	0.989	-	0.073 (0.116–0.123)	-	0.026
M1	22.948	14	3.898	4	0.061	0.985	0.989	0.000	0.061 (0.000–0.105)	−0.012	0.042
M2	35.114	18	12.166	4	0.009	0.977	0.980	−0.009	0.075 (0.037–0.112)	0.014	0.051
M3	44.662	23	9.548	5	0.004	0.978	0.974	−0.006	0.075 (0.041–0.107)	0.000	0.055

Note: number of observations per group: Asian international students = 167; Italian students = 171. ^a^ Models followed a sequential constraint imposition. The analyses started with the least constrained model and subsequent restrictions include the restriction imposed in the preceding model. M0 = configural; M1 = metric; M2 = scalar; M3 = strict.

## Data Availability

The data used to support the findings of this study are available upon request from the corresponding author.

## Appendix A

**Table A1:** Inter-item correlations of the SWLS items.

Item	SWLS_1	SWLS_2	SWLS_3	SWLS_4	SWLS_5
SWLS_1	-	0.645	0.765	0.570	0.488
SWLS_2	0.671	-	0.698	0.447	0.324
SWLS_3	0.733	0.689	-	0.617	0.492
SWLS_4	0.553	0.500	0.689	-	0.431
SWLS_5	0.487	0.426	0.527	0.485	-

Note: correlations between items of the English version of the SWLS for Asians are shown under the diagonal; correlations between items of the Italian version of the SWLS are shown above the diagonal. All correlations are significant at *p <* 0.001.

## References

[1] Diener E., Emmons R., Larsen R., Griffin S. (1985). The Satisfaction with Life Scale. J. Personal. Assess..

[2] Pavot W., Diener E. (2008). The satisfaction with life scale and the emerging construct of life satisfaction. J. Posit. Psychol..

[3] Garrido S., Méndez I., Abellán J.-M. (2013). Analysing the simultaneous relationship between life satisfaction and health-related quality of life. J. Happiness Stud..

[4] Habibov N., Afandi E. (2016). Does life satisfaction determine subjective health?. Appl. Res. Qual. Life.

[5] Milas G., Martinović Klarić I., Malnar A., Saftić V., Šupe-Domić D., Slavich G.M. (2021). The impact of stress and coping strategies on life satisfaction in a national sample of adolescents: A structural equation modelling approach. Stress Health.

[6] Szcześniak M., Mazur P., Rodzeń W., Szpunar K. (2021). Influence of life satisfaction on self-esteem among young adults: The mediating role of self-presentation. Psychol. Res. Behav. Manag..

[7] Karaman M.A., Watson J.C. (2017). Examining associations among achievement motivation, locus of control, academic stress, and life satisfaction: A comparison of US and international undergraduate students. Personal. Individ. Differ..

[8] Pavot W., Diener E., Suh E. (1998). The temporal satisfaction with life scale. J. Personal. Assess..

[9] Busseri M.A., Choma B.L., Sadava S.W. (2009). “As good as it gets” or “The best is yet to come”? How optimists and pessimists view their past, present, and anticipated future life satisfaction. Personal. Individ. Differ..

[10] Bai X., Wu C., Zheng R., Ren X. (2011). The psychometric evaluation of the Satisfaction with Life Scale using a nationally representative sample of China. J. Happiness Stud..

[11] Wu C.-H., Yao G. (2006). Analysis of factorial invariance across gender in the Taiwan version of the Satisfaction with Life Scale. Personal. Individ. Differ..

[12] Bacro F., Coudronnière C., Gaudonville T., Galharret J.-M., Ferrière S., Florin A., Guimard P. (2020). The French adaptation of the Satisfaction with Life Scale (SWLS): Factorial structure, age, gender and time-related invariance in children and adolescents. Eur. J. Dev. Psychol..

[13] Esnaola I., Benito M., Agirre I.A., Freeman J., Sarasa M. (2017). Measurement invariance of the Satisfaction with Life Scale (SWLS) by country, gender and age. Psicothema.

[14] Moreta-Herrera R., Perdomo-Pérez M., Reyes-Valenzuela C., Gavilanes-Gómez D., Rodas J.A., Rodríguez-Lorenzana A. (2023). Analysis from the classical test theory and item response theory of the Satisfaction with Life Scale (SWLS) in an Ecuadorian and Colombian sample. J. Hum. Behav. Soc. Environ..

[15] Zanon C., Bardagi M.P., Layous K., Hutz C.S. (2014). Validation of the Satisfaction with Life Scale to Brazilians: Evidences of measurement noninvariance across Brazil and US. Soc. Indic. Res..

[16] Wang D., Hu M., Xu Q. (2017). Testing the factorial invariance of the Satisfaction with Life Scale across Chinese adolescents. Soc. Behav. Personal. Int. J..

[17] Arrindell W.A., Checa I., Espejo B., Chen I.-H., Carrozzino D., Vu-Bich P., Dambach H., Vagos P. (2022). Measurement invariance and construct validity of the Satisfaction with Life Scale (SWLS) in community volunteers in Vietnam. Int. J. Environ. Res. Public Health.

[18] Hultell D., Gustavsson J.P. (2008). A psychometric evaluation of the Satisfaction with Life Scale in a Swedish nationwide sample of university students. Personal. Individ. Differ..

[19] Wu C.-H., Chen L.H., Tsai Y.-M. (2009). Longitudinal invariance analysis of the Satisfaction With Life Scale. Personal. Individ. Differ..

[20] Whisman M.A., Judd C.M. (2016). A cross-national analysis of measurement invariance of the Satisfaction with Life Scale. Psychol. Assess..

[21] Valenti G.D., Magnano P., Faraci P. (2022). Evaluating the Dimensionality of the Sociocultural Adaptation Scale in a Sample of International Students Sojourning in Los Angeles: Which Difference between Eastern and Western Culture?. Eur. J. Investig. Health Psychol. Educ..

[22] Harzing A.-W. (2006). Response styles in cross-national survey research: A 26-country study. Int. J. Cross Cult. Manag..

[23] Jang S., Kim E.S., Cao C., Allen T.D., Cooper C.L., Lapierre L.M., O’Driscoll M.P., Sanchez J.I., Spector P.E., Poelmans S.A. (2017). Measurement invariance of the satisfaction with life scale across 26 countries. J. Cross-Cult. Psychol..

[24] Schnettler B., Miranda-Zapata E., Lobos G., del Carmen Lapo M., Adasme-Berríos C., Hueche C. (2017). Measurement invariance in the Satisfaction with Life Scale in Chilean and Ecuadorian older adults. Personal. Individ. Differ..

[25] Jovanović V., Rudnev M., Arslan G., Buzea C., Dimitrova R., Góngora V., Guse T., Ho R.T., Iqbal N., Jámbori S. (2022). The Satisfaction with Life Scale in adolescent samples: Measurement invariance across 24 countries and regions, age, and gender. Appl. Res. Qual. Life.

[26] Brown T.A. (2015). Confirmatory Factor Analysis for Applied Research.

[27] Cook R.M. (2021). Addressing missing data in quantitative counseling research. Couns. Outcome Res. Eval..

[28] Diener E. (1984). Subjective well-being. Psychol. Bull..

[29] Di Fabio A., Busoni L. (2009). Proprietà psicometriche della versione italiana della Satisfaction with Life Scale (SWLS) con studenti universitari. Couns. G. Ital. Ric. Appl..

[30] Cain M.K., Zhang Z., Yuan K.-H. (2017). Univariate and multivariate skewness and kurtosis for measuring nonnormality: Prevalence, influence and estimation. Behav. Res. Methods.

[31] Hu L.t., Bentler P.M. (1999). Cutoff criteria for fit indexes in covariance structure analysis: Conventional criteria versus new alternatives. Struct. Equ. Model. A Multidiscip. J..

[32] Marsh H.W., Hau K.-T., Grayson D., Maydeu-Olivares A., McArdle J.J. (2005). Goodness of fit in structural equation models. Contemporary Psychometrics: A Festschrift for Roderick P. McDonald.

[33] Marsh H.W., Hau K.-T., Wen Z. (2004). In search of golden rules: Comment on hypothesis-testing approaches to setting cutoff values for fit indexes and dangers in overgeneralizing Hu and Bentler’s (1999) findings. Struct. Equ. Model..

[34] Hayes A.F., Coutts J.J. (2020). Use omega rather than Cronbach’s alpha for estimating reliability. But…. Commun. Methods Meas..

[35] Cheung G.W., Rensvold R.B. (2002). Evaluating goodness-of-fit indexes for testing measurement invariance. Struct. Equ. Model..

[36] Chen F.F. (2007). Sensitivity of goodness of fit indexes to lack of measurement invariance. Struct. Equ. Model. A Multidiscip. J..

[37] Di Fabio A., Gori A. (2016). Measuring adolescent life satisfaction: Psychometric properties of the satisfaction with life scale in a sample of Italian adolescents and young adults. J. Psychoeduc. Assess..

